# HoxA9 regulated Bcl-2 expression mediates survival of myeloid progenitors and the severity of HoxA9-dependent leukemia

**DOI:** 10.18632/oncotarget.1306

**Published:** 2013-09-15

**Authors:** Gabriela Brumatti, Marika Salmanidis, Chung H Kok, Rebecca A Bilardi, Jarrod J Sandow, Natasha Silke, Kylie Mason, Jolanda Visser, Anissa M Jabbour, Stefan P Glaser, Toru Okamoto, Philippe Bouillet, Richard J D'Andrea, Paul G Ekert

**Affiliations:** ^1^ Walter and Eliza Hall Institute of Medical Research, 1G Royal Parade, Parkville, Australia; ^2^ Department of Medical Biology, University of Melbourne, Parkville, Australia; ^3^ Murdoch Children's Research Institute and Children's Cancer Centre, Royal Children's Hospital, Parkville, Australia; ^4^ Department of Pediatrics, University of Melbourne, Parkville, Australia; ^5^ Department of Medicine, University of Melbourne, Parkville, Australia; ^6^ Department of Hematology and Centre for Cancer Biology, SA Pathology, Adelaide; Department of Hematology and Oncology, the Queen Elizabeth Hospital, Woodville, Australia; The Discipline of Medicine, University of Adelaide; ^7^ University Medical Center Groningen, Hanzeplein 1, Groningen, Netherland; ^8^ Department of Molecular Virology, Research Institute for Microbial Diseases, Osaka University, 3-1 Yamadaoka, Suita, Osaka, Japan

**Keywords:** HoxA9, Bcl-2, Leukemia, apoptosis

## Abstract

Deregulated expression of *Hox* genes such as HoxA9 is associated with development of myeloproliferative disorders and leukemia and indicates a poor prognosis. To investigate the molecular mechanisms by which HoxA9 promotes immortalization of hematopoietic cells, we generated growth factor dependent myeloid cells in which HoxA9 expression is regulated by administration of 4-hydroxy-tamoxifen. Maintenance of HoxA9 overexpression is required for continued cell survival and proliferation, even in the presence of growth factors. We show for the first time that maintenance of Bcl-2 expression is critical for HoxA9-dependent immortalization and influences the latency of HoxA9-dependent leukemia. Hematopoietic cells lacking Bcl-2 were not immortalized by HoxA9 *in vitro*. Furthermore, deletion of Bcl-2 delayed the onset and reduced the severity of HoxA9/Meis1 and MLL-AF9 leukemias. This is the first description of a molecular link between HoxA9 and the regulation of Bcl-2 family members in acute myeloid leukemia.

## INTRODUCTION

The Hox genes are a family of highly conserved transcriptional regulators with critical roles in normal development and hematopoiesis [1-3]. Elevated expression of HoxA9 is strongly and causally associated with the development of myeloid leukemia and correlates with a poor prognosis [4]. HoxA9 overexpression frequently occurs in association with rearrangements of the Mixed Lineage Leukemia locus at 11q23 (MLL) [5,6], but is also associated with trisomy 8 AML [7] and the t(7;11)(p15;p15) translocation which fuses NUP98 to HoxA9 [8]. Transformation of myeloid progenitor cells by transgenic expression of MLL rearrangements is dependent on the expression of HoxA group genes, in particular HoxA9 [9,10].

HoxA9 overexpression maintains the self-renewal capacity of leukemic stem cells and blocks differentiation [11,12], inducing a gene-expression signature associated with hematopoietic self-renewal potential [6]. HoxA9 regulates transcription in complex with homeodomain-containing proteins of the Pre-B cell/CEH-20 family (PBC proteins, Pbx proteins in vertebrates) and the Meis family, particularly Meis1 [13-15]. Hox-Pbx binding requires a hexapeptide motif containing a critical tryptophan residue in the Hox proteins [16]. Mutation of the hexapeptide domain of HoxA9 abolishes its activity to immortalize myeloid cells [11].

Chromatin-immunoprecipitation and sequencing (ChIP-seq), combined with expression microarrays, have mapped potential HoxA9-regulated genes [17,18]. These studies suggest HoxA9 functions as a transcriptional enhancer, which together with Meis 1 and lineage-restricted transcription factors, activate leukemia-associated proto-oncogenes that include *Myb* and *Flt3* [17].

Several lines of evidence indicate that HoxA9 regulates pathways that control cell survival. For example, increased apoptosis is observed in the fetal thymus of *HoxA9*^−/−^ mice [3] and silenced HoxA9 expression induces apoptosis in human AML lines bearing MLL rearrangements [18], suggesting that one function of HoxA9 is to repress apoptosis pathways.

To investigate how HoxA9 regulates cell viability, we used a lentiviral expression system to express HoxA9 in myeloid progenitor cells under the control of a 4-hydroxy-tamoxifen (4-OHT) inducible promoter. Downregulation of HoxA9 expression induced apoptosis in the majority of cells and was correlated with the loss of Bcl-2 expression. Moreover, deletion of Bcl-2 expression in HoxA9 factor-dependent cells induced apoptosis. In HoxA9/Meis1 and MLL-AF9-dependent AML, deletion of Bcl-2 dramatically altered clinical features, resulting in longer survival, diminished peripheral white cell counts and more destructive splenic disease. Together our data show that HoxA9 regulated Bcl-2 expression is important for the survival of myeloid progenitors and contributes to the oncogenic effects of HoxA9 overexpression.

## RESULTS

### HoxA9 expression is required for survival and clonogenic proliferation of IL-3 and GM-CSF dependent myeloid progenitor cells

**Figure 1:**
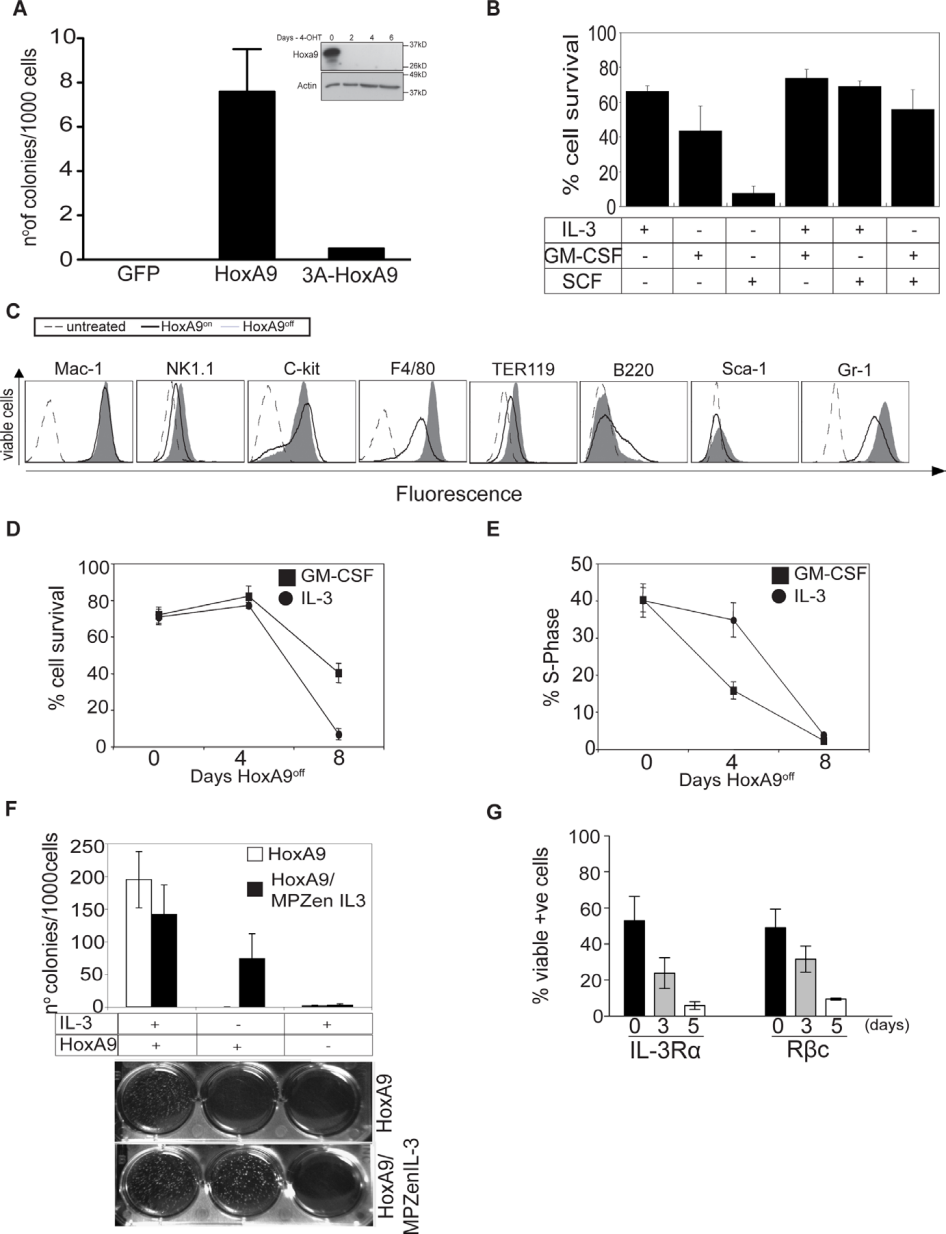
HoxA9 overexpression is required for proliferation and survival of growth factor dependent myeloid cells (A) Hematopoietic progenitor cells were transduced with GFP, Flag-HoxA9 or Flag-hexapeptide mutant HoxA9 and cultured in soft agar in the presence of the IL-3 (0.5ng/ml) and GM-CSF (2.5ng/ml). After 14 days, number of colonies was counted and the results expressed as colonies per 1000 cells plated. Results are means ±SEM of 3 independent experiments using 3 independent pools of cells. Inset shows lysates from c-kit+/Lin-myeloid precursors immortalized with inducible Flag-tagged HoxA9, cultured in the presence (Day 0) or absence of 4-OHT probed with an anti-HoxA9 and anti-Actin antibodies as a loading control. (B) Viability of HoxA9 FDM cells cultured in the presence of the indicated cytokines for 48 hours was determined by flow cytometric measurement of AnnexinV staining and PI exclusion. Results show the means ± SEM of 4 independent experiments. (C) HoxA9 FDM cells maintained in GM-CSF were cultured in the presence or absence of 4-OHT for 4 days. Cells were stained with antibodies to the indicated surface lineage markers and expression assessed by flow cytometry. Unstained cells represented by the dashed line act as a control. Cells expressing HoxA9 (HoxA9^on^) are represented by the solid line and cells in which HoxA9 has been downregulated (HoxA9^off^) are indicated by the filled histogram. Representative histograms are shown. (D) The viability of HoxA9 FDM cells was determined using flow cytometric determination of AnnexinV-FITC staining and PI exclusion, at 0, 4 and 8 days following removal of 4-OHT from cultures to downregulate HoxA9 expression. Results are means ±SEM of 4 clones repeated in 2 independent experiments. (E) HoxA9 FDM cells were cultured in the absence of 4-OHT for 8 days. At the indicated time points, cells were stained using a hypotonic PI buffer solution and the percentage of cells in S phase determined by flow cytometry. The results are means ±SEM of 4 clones repeated 2 independent experiments. (F) HoxA9 FDM cells were infected with retrovirus encoding IL-3 or empty control virus. Infected cells were plated in soft agar in the presence or absence of 4-OHT and presence or absence of exogenous IL-3. The number of colonies per 1000 cells plated was determined after 10 days in culture. Results show means ±SEM of 3 independent experiments using 2 independent pools of HoxA9 infected cells. Representative example of colony formation in the assays is shown below. (G) HoxA9 FDM cells cultured in the presence of IL-3 and GM-CSF were stained with antibodies to detect the indicated components of the IL-3 receptor 3 and 5 days after 4-OHT deprivation. Expression was analyzed by flow cytometry. Data are means ±SEM of 3 independent experiments from 4 independent pools of HoxA9 infected cells.

HoxA9 overexpression immortalizes myeloid progenitor cells dependent on cytokines such as IL-3 or GM-CSF. To explore the consequences of varying HoxA9 expression, we infected c-kit^+^/lin^−^ myeloid progenitor cells with lentivirus encoding a Flag-tagged HoxA9 under the control of a 4-OHT-inducible promoter to generate HoxA9 Factor Dependent Myeloid (FDM) cells. For simplicity, we refer to the 4-OHT-induced expression of HoxA9 as HoxA9^on^, and the absence of 4-OHT as HoxA9^off^. Consistent with previous results [11], overexpression of Flag-HoxA9, but not Flag-HoxA9 lacking the hexapeptide domain, efficiently immortalized myeloid progenitor cells (Figure 1A), even with 10 fold higher cytokine concentrations (Supplementary Figure S1A). We tested various cytokine combinations to determine which ones maintain HoxA9 FDM cell viability (Figure 1B). Only IL-3 or GM-CSF or the combination of those kept cells alive. In contrast, Stem Cell Factor (SCF) was insufficient to maintain cell survival. Cytokine removal resulted in rapid apoptosis, indicating that these cells were strictly cytokine-dependent (Supplementary Figure S1B). We then compared the expression of surface lineage markers in HoxA9^on^ and HoxA9^off^ cells cultured in GM-CSF. In the HoxA9^off^ state, a proportion of cells differentiated, as described by others [11], indicated by increased F4/80 (macrophage/monocyte marker) and Gr-1 (granulocyte marker) expression (Figure 1C).

The majority of HoxA9^off^ cells were refractory to the proliferative signals transduced by IL-3 or GM-CSF, resulting in declined cell viability after 4 days in HoxA9^off^ conditions (Figure 1D, Supplementary Figure S1A-C). This effect was more pronounced in cells cultured in IL-3 compared to GM-CSF. Loss of viability was preceded by a decline in the proportion of cells in S-phase (Figure 1E). Clonogenic assays showed that HoxA9^off^ FDM cells failed to form colonies, even in 10-fold higher concentrations of IL-3 or GM-CSF (Supplementary Figure S1C). Importantly, the kinetics of cell death in response to HoxA9^off^ was slower than that induced by cytokine deprivation (compare Figure 1D to Supplementary Figure S1B). Together these data demonstrate that the loss of HoxA9 expression induced cells to stop dividing and undergo apoptosis.

We next determined whether loss of HoxA9 expression also prevented colony formation in cells that endogenously produce cytokines. HoxA9 FDM cells infected with a retrovirus encoding IL-3 or a control virus were cultured in soft agar in the presence or absence of IL-3 and HoxA9 (Figure 1F). Endogenously produced, or exogenously provided IL-3 promoted colony formation in HoxA9^on^ but not HoxA9^off^ cells, demonstrating that autonomous IL-3 signaling is sufficient to permit proliferation and viability of hematopoietic progenitor cells only provided HoxA9 expression is maintained.

It has been shown in HoxA9-immortalized hematopoietic cells that HoxA9 downregulation results in decreased transcription of IL-3 receptor α chain (IL-3α or CD123), β common chain (βc or CD131), CD135 (Flt3) and reduced CD34 expression [17]. We determined the levels of these markers in multiple independent lines in HoxA9^on^ and HoxA9^off^ (Figure 1G and Supplementary Figure S1D). The expression of IL-3Rα, βc and CD34 protein always decreased in HoxA9^off^ cells, while CD135 levels remained virtually undetectable. These data indicate that once HoxA9 is turned off, the expression of the IL-3/GM-CSF receptors declines, which may contribute to the unresponsiveness to these cytokines.

### Altering HoxA9 expression changes Bcl-2 expression levels

**Figure 2:**
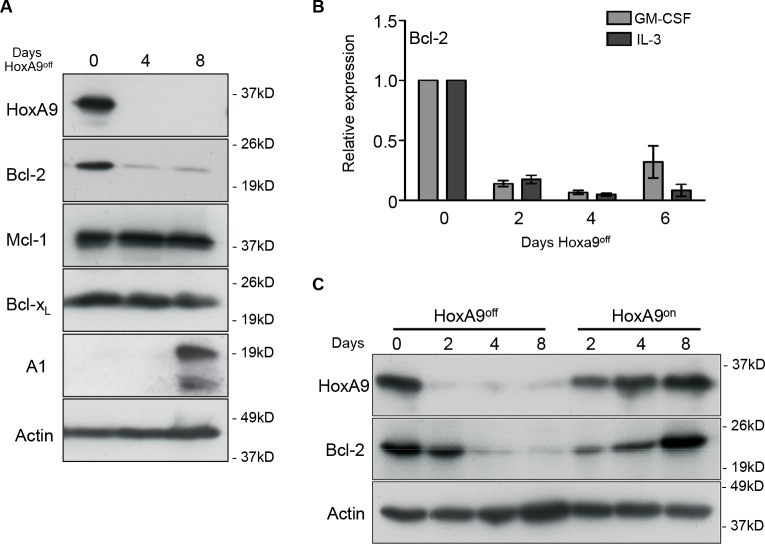
Downregulated HoxA9 expression results in loss of Bcl-2 expression (A) HoxA9 FDM cells were cultured in media containing GM-CSF and IL-3 in the presence (Day 0) or absence of 4-OHT (HoxA9^off^) over 8 days. At the indicated times, lysates were harvested and Western blots probed with antibodies against HoxA9, Bcl-2, Mcl-1 and Bcl-x_L_, Actin was used as a loading control. (B) Bcl-2 mRNA levels were determined by qRT-PCR using total RNA derived from HoxA9 FDM cells cultured in the presence of cytokines and absence of 4-OHT for 6 days. mRNA levels of Bcl-2 were normalized to Sdh2a and Polr2a. Results show mean ± SE from 4 independent pools or clones each of IL-3- and GM-CSF-dependent FDM cells repeated in 2 independent experiments. (C) HoxA9 FDM cells, maintained in IL-3 and GM-CSF, were cultured without 4-OHT (HoxA9^off^) followed by 4-OHT re-addition (HoxA9^on^). Lysates were generated at the indicated time points and Western blots probed with antibodies against HoxA9 and Bcl-2. Anti-actin antibody was used as a loading control.

Apoptosis mediated by IL-3 withdrawal is regulated by members of the Bcl-2 family of proteins [18]. Since loss of HoxA9 made cells refractory to survival signals transduced by IL-3, we looked for changes in anti-apoptotic Bcl-2 family member expression in response to turning off HoxA9 expression (Figure 2A). We consistently observed reduced Bcl-2 protein expression in cell lines in the HoxA9^off^ state, whereas Mcl-1 and Bclx_L_ expression remained unchanged and A1 expression was increased. Consistent with the changes in protein expression, Bcl-2 mRNA decreased in HoxA9^off^ cells (Figure 2B). When HoxA9 expression was turned back on, Bcl-2 returned to levels similar to those prior to removal of 4-OHT (Figure 2C), emphasizing a direct correlation between induced HoxA9 expression and Bcl-2 levels. We looked for conserved HoxA9/Meis1 binding in Bcl-2 family members using previously published Chip-sequence data (GSE38339 and GSE33518). Only Bcl-2 showed conserved HoxA9 binding consistent with other validate HoxA9 targets (Supplemental Figure 4B-D)

**Figure 3:**
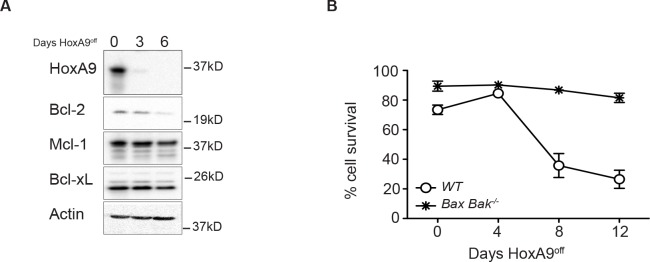
Decline in Bcl-2 expression in HoxA9-dependent myeloid cells is independent of apoptotic cell death (A) The viability of wild type and *Bax;Bak*^−/−^ HoxA9 FDM cells was determined by flow cytometry analysis of AnnexinV-FITC staining and PI exclusion, at 0, 4, 8 and 12 days following removal of 4-OHT. Results are means ±SEM of 2 pools and 2 clones repeated in 3 independent experiments. (B) *Bax;Bak*^−/−^ HoxA9 immortalized cells were cultured in the presence (Day 0) or absence of 4-OHT over 6 days. At the indicated times, lysates were harvested and Western blots probed with antibodies against HoxA9, Bcl-2, Mcl-1 and Bcl-x_L_. Actin was used as a loading control.

To determine if the diminished Bcl-2 expression was due to cell loss via apoptosis, we immortalized hematopoietic progenitor cells from mice lacking the key apoptosis regulators Bax and Bak. These cells are resistant to apoptosis induced by cytokine deprivation [19]. *Bax*^−/−^;*Bak*^−/−^ HoxA9^off^ FDM cells remained viable, however Bcl-2 expression still decreased (Figure 3A and B), showing that varying HoxA9 expression resulted in concomitant changes in Bcl-2 expression.

We speculated that changes in Bcl-2 expression following HoxA9 downregulation would alter the sensitivity of HoxA9 FDM cells to chemotherapeutic agents. However despite the changes in Bcl-2 family member expression in HoxA9^on^ or HoxA9^off^ conditions, the susceptibility to apoptosis induced by chemotherapeutic drugs was not altered. This included the BH3 mimetic drug ABT-737 (Supplementary Figure S2A and B). Mcl-1 expression was not affected by HoxA9 expression, probably accounting for the unchanged sensitivity to ABT-737.

### Bcl-2 is required for HoxA9-dependent immortalization *in vitro*

**Figure 4:**
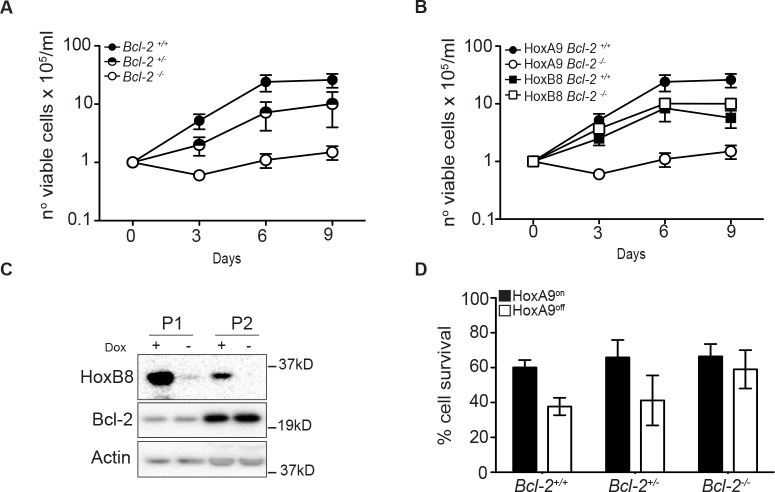
Bcl-2 is required for HoxA9-dependent immortalization of myeloid cells (A-B) Hematopoietic progenitor cells from *Bcl-2*^+/+^, *Bcl-2*^+/−^ and *Bcl-2*^−/−^ littermates were infected with HoxA9 or HoxB8 and selected for 10 days in media containing IL-3 (0.5ng/ml) (HoxB8) or IL-3 and GM-CSF (2.5ng/ml) (HoxA9) and 0.5ug/ml puromycin. Cells were then replated in liquid culture at several known dilutions (day 0) and cell number determined at the indicated days and expressed as a value relative to the number of cells plated on day 0. HoxA9-immortalized cells data are means ±SEM from 5 independently infected pools of *Bcl-2*^+/+^, 6 independent pools of *Bcl-2*^+/−^ and 7 independent pools of *Bcl-2*^−/−^ performed in 3 independent experiments. HoxB8-immortalized cells results are means ±SEM from 3 independent experiments of 3 independent pools from each genotype. (C) Hematopoietic stem cells from wild type mice were immortalized with lentivirus encoding HoxB8 under the control of a tetracycline-inducible promoter. Cells were cultured in the presence or absence of 1.5μg/ml doxycycline and lysates probed with antibodies to detect Bcl-2, HoxB8 and Actin as a loading control. Two independent pools (P1 and P2) are shown. (D) Hematopoietic cells derived from *Bcl-2*^+/+^, *Bcl-2*^−/+^ and *Bcl-2*^−/−^ mice, infected with HoxA9 lentivirus, were cultured in the presence (HoxA9^on^) or absence (HoxA9^off^) of 4-OHT. Viability was then determined by flow cytometry analysis of AnnexinV-FITC staining and PI exclusion. Results are means ±SEM of 3 independent clones from each genotype repeated in 2 independent experiments.

We next addressed the role of Bcl-2 in HoxA9-dependent immortalization of myeloid progenitor cells by attempting to generate HoxA9 FDM cells from wild-type (*Bcl-2*^+/+^), *Bcl-2*^+/−^ and *Bcl-2*^−/^^−^ mice. After transduction with HoxA9 lentivirus and selection for infection, equal cell numbers of *Bcl-2*^+/+^, *Bcl-2*^+/−^ and *Bcl-2*^−/−^ cells were seeded in liquid culture containing cytokines and cell numbers were assessed over 9 days. The numbers of wild-type and *Bcl-2*^+/−^ cells increased steadily, consistent with HoxA9-dependent immortalization (Figure 4A). The plateau in the rate of proliferation by day 9 was a result of cells exiting log phase growth due to high cell numbers in the culture dishes. The numbers of *Bcl-2*^−/−^ HoxA9 infected cells did not increase in the same time period (Figure 4A). To exclude the possibility that progenitor cells from *Bcl-2*^−/−^ mice generally fail to be immortalized by Hox genes, we compared the ability of HoxA9 and HoxB8 to generate FDM cells. Equal cell numbers of wild type and *Bcl-2*^−/−^ cells were infected with lentivirus of matched MOI encoding HoxA9 or HoxB8 and cell numbers were assessed over a 9 days period (Figure 4B). HoxB8 and HoxA9 infection induced rapid proliferation of wild type cells but only HoxB8 induced proliferation of *Bcl-2*^−/−^ cells and permitted the establishment of cell lines. HoxB8 immortalized *Bcl-2*^−/−^ cells expressed similar surface lineage markers as wild type cells (Supplementary Figure S3A). Moreover, turning off HoxB8 expression in wild type cells did not induce changes in Bcl-2 expression as observed in HoxA9 cells (Figure 4C). These data indicate that in contrast to HoxB8, HoxA9 requires Bcl-2 to immortalize cytokine dependent myeloid cells.

Eventually, cells did grow out from liquid cultures of HoxA9-infected *Bcl-2*^−/−^ cells or the rare colony formed in soft-agar. These cells differed from HoxA9-immortalized wild type cells. A much higher proportion of *Bcl-2*^−/−^ cells expressed the natural killer cell marker NK1.1 (Supplementary Figure S3B). Importantly, the viability of these surviving *Bcl-2*^−/−^ cells was less dependent on HoxA9 expression (Figure 4D). These results confirm that the rapid immortalization of progenitor cells by HoxA9 required the presence of at least one copy of Bcl-2.

**Figure 5:**
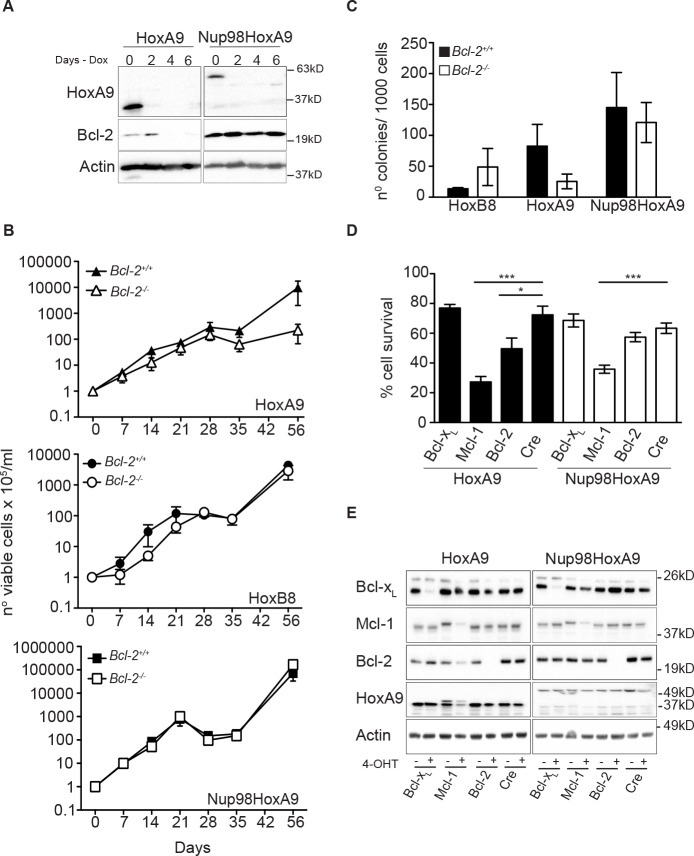
HoxA9-dependent hematopoietic cells but not Nup98-HoxA9 or HoxB8, requires Bcl-2 expression for immortalization and survival (A) Hematopoietic progenitor cells were immortalized by infection with lentivirus encoding HoxA9 or Nup98-HoxA9 under the control of a tetracycline-repressible promoter. Cells were cultured in the absence (Day 0) or presence of 1.5μg/ml doxycycline for the indicated time course. Lysates were harvested and probed with antibodies against HoxA9, Bcl-2 and actin was used as a loading control. (B) Hematopoietic progenitor cells derived from *Bcl-2*^+/+^ and *Bcl-2*^−/−^ were infected with HoxA9, HoxB8 or Nup98-HoxA9 and cultured in the presence of IL-3 (5ng/ml) alone (HoxB8) or IL-3 and GM-CSF (10ng/ml) (HoxA9 and Nup98-HoxA9). Seven days after infection, cells were replated at 2.5 × 10^5^ cells/ml or 5 × 10^5^ cells/ml and counted at the indicated time points over a 56 day time course. Cells were passage at known dilutions every 3-4 days. Results are means ±SEM from 3 independent experiments of 6 independent pools from each genotype. (C) At day 42 of the experiments described in (B), cells were plated in 0.3% soft agar in the presence of cytokines and 4-OHT. Colonies were counted after 14 days. Results are means ±SEM of 3 independent experiments of 6 independent pools from each genotype. (D) Hematopoietic progenitor cells from mice with homozygous LoxP-flanked Bcl-2, Mcl-1 or Bcl-xL alleles crossed with Rosa:Cre-ER were infected with HoxA9 (filled bars) or Nup98-HoxA9 (unfilled bars) and cultured in IL-3 and GM-CSF. Cells were then treated with 65nM of 4-OHT to induce deletion of the LoxP allele and viability measures using flow cytometry measurement of Annexin V-FITC staining and PI exclusion 72 hours later. Results show means ±SEM of 5 independent experiments from HoxA9 FDM cells and 4 independent experiments from Nup98-HoxA9 FDMs. (*) P <0.05 and (***) P <0.0005. (E) Western blot of representative pools of cells from (D) probed with antibodies against Bcl-2, Bcl-x_L_ and Mcl-1. Note the conditional allele of Mcl-1 runs at a higher MW than the endogenous allele.

The t(7:11)(p15:p15) translocation, which fuses the nucleoporin *Nup98* to the homeodomain of *HoxA9*, has been identified in AML patients. To investigate whether deletion of Bcl-2 also impaired immortalization of myeloid cells by Nup98-HoxA9, hematopoietic progenitor cells were infected with lentivirus encoding Nup98-HoxA9 under the control of a tetracycline-inducible promoter. Compared to HoxA9 tetracycline-inducible FDMs, expression of both HoxA9 and Nup98-HoxA9 was repressed by the removal of Doxycycline from the media (Figure 5A) however, only HoxA9 cells presented changes on Bcl-2 expression. We then infected hematopoietic progenitor cells from *Bcl-2*^+/+^ and *Bcl-2*^−/−^ mice with HoxA9, Nup98-HoxA9 or HoxB8 and maintained cells in high concentration of cytokines. Cells were replated at equal numbers 7 days after infection (Day 0) and counted over 56 days. Nup98-HoxA9 immortalized wild-type and *Bcl-2*^−/−^ cells with similar efficiency (bottom panel Figure 5B). HoxA9-infected *Bcl-2*^−/−^ cells proliferated at the same rate as wild-type cells for up to 28 days, after which proliferation ceased. This was in contrast of the previous results and shows that in extended assays, some initial proliferation of *Bcl-2*^−/−^ cells will occur (top panel Figure 5B compare to Figure 4A and B). One critical distinction between the short-term (Figure 4A and B) and the long-term (Figure 5B) assays is that cells in Figure 4 have been selected in puromycin and maintained in culture for up to 30 days before the assays were performed, therefore bypassing the initial proliferation phase, which explains the different patterns of proliferation observed in these experiments. The period of initial proliferation, in the absence of selection, of Bcl-2−/− cells in the first 28 days (Figure 5B) corresponds to the time taken for puromycin selection of cells shown in Figure 4A and B. As before, HoxB8 infection immortalized *Bcl-2*^−/−^ progenitor cells (middle panel Figure 5B). At 21 and 42 days, cells were removed from these cultures and plated at equal density in soft agar and the number of colonies was counted. No significant difference was observed in the colony numbers of *Bcl-2*^+/+^ and *Bcl-2*^−/−^ progenitor cells immortalized with HoxB8 or Nup98-HoxA9 (Figure 5C and Supplementary Figure S3C). However, decreased colony formation in HoxA9-infected *Bcl-2*^−/−^ cells was observed. These data show that the requirement for Bcl-2 in the immortalization of progenitor cells is waived when cells are infected with Nup98-HoxA9. This was further supported by the findings that in the presence or absence of Nup98-HoxA9 expression, Bcl-2 expression was unchanged (Figure 5A).

Having shown that Bcl-2 was required for HoxA9-dependent immortalization, we next asked whether this requirement persisted after cells were immortalized. We harvested bone marrow from mice homozygous for Bcl-x_L_, Mcl-1 or Bcl-2 alleles flanked by LoxP sites [20-22] that had been crossed with syngeneic mice expressing cre-recombinase fused to a modified estrogen receptor tag (Rosa26:Cre-ERT2) expressed from the Rosa 26 locus [23]. Bone marrow stem cells from these animals were immortalized by infection with virus encoding constitutively expressed HoxA9 or Nup98-HoxA9, in the continuous presence of IL-3 and GM-CSF. Once continuously proliferating, 4-OHT was added to cultures to induce deletion of *Bcl-x_L_, Mcl-1* or *Bcl-2* and viability was determined (Figure 5D). Western blot demonstrated efficient deletion of the LoxP-flanked alleles (Figure 5E). Deletion of *Mcl-1* reduced viability in both HoxA9 and Nup98-HoxA9 infected cells, consistent with the published role for Mcl-1 in the survival of hematopoietic cells [24] (Figure 5D and Supplementary Figure S3D). Deletion of *Bcl-2* reduced the viability of HoxA9-immortalized cells, but not of Nup98-HoxA9 cells (Figure 5D). Deletion of *Bcl-x_L_* had no significant impact on cell viability of either sets of cells. Together, these data provide further evidence that Bcl-2 expression is specifically required to maintain cytokine-dependent viability of HoxA9-immortalized cells.

### 11q23 rearrangements are associated with increased Bcl-2 expression

**Figure 6:**
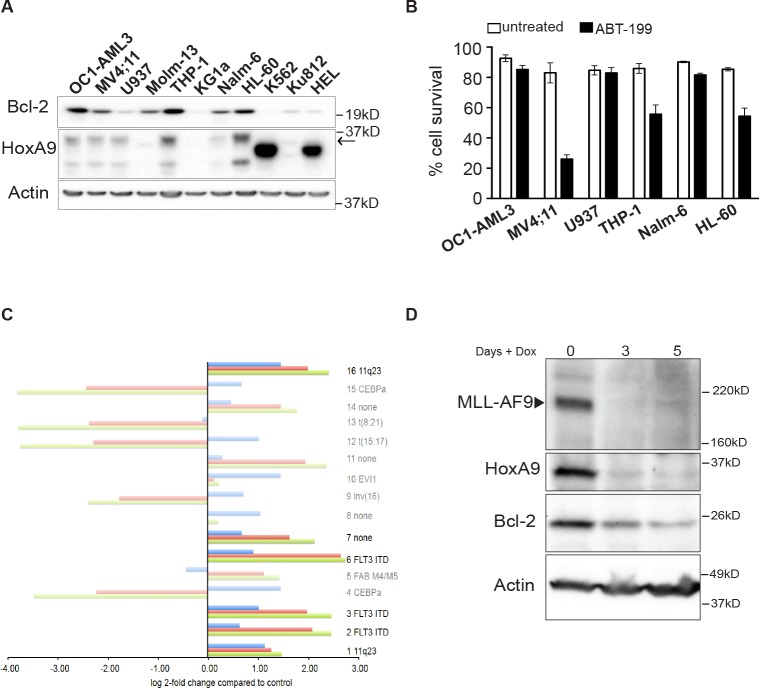
Association between HoxA9 and Bcl-2 expression in human leukemic lines (A) Expression of HoxA9 and Bcl-2 in human leukemic lines. Total cell lysates were harvested and probed with antibodies against HoxA9, Bcl-2 and actin was used as a loading control. * Unknown band, which may be a HoxA9 splice variant. (B) Sensitivity of human leukemic lines expressing HoxA9 to Bcl-2 inhibitor ABT-199. Human leukemic cells expressing higher levels of endogenous HoxA9 were treated or not with 1μM of ABT199 for 48hs. Cell survival was determined by flow cytometric analysis of PI exclusion. Results show means ±SEM of 3 independent experiments. (C) Expression of HoxA9 (2 probesets – 209905_at and 214651_s_at) and Bcl-2 (one probeset – 203685_at) from 285 AML samples grouped into each of 16 clusters characterized by gene expression profiles previously defined by Valk et al [29] (GSE1159). Where a cluster is also associated with a particular genetic lesion, this is shown on the right of each cluster and where no such association exists this is shown as “none”. Bright bars indicate the clusters characterized by significant association between HoxA9 and Bcl-2 expression. Expression for each cluster is shown relative to the normal control group (NBM and CD34+, n=8). Significant genes were defined as BH-adjusted p<0.05. (D) Expression of MLL-AF9, HoxA9 and Bcl-2 in MLL-AF9 immortalized cell lines. Hematopoietic progenitor cells were immortalized by infection with lentivirus encoding MLL-AF9 under the control of a tetracycline-repressible promoter. Cells were cultured in the absence (Day 0) or presence of 100ng/ml doxycycline for 3 and 5 days. Lysates were harvested and probed with antibodies against MLL, HoxA9, Bcl-2 and actin was used as a loading control.

In human AML, elevated HoxA9 expression is strongly associated with rearrangements of the Mixed Lineage Leukemia locus (11q23 or MLL) [25-28]. To explore a possible association between HoxA9 and Bcl-2 expression in human AML cell lines and patient samples, we probed lysates from a range of leukemic cell lines for HoxA9 and Bcl-2 expression (Figure 6A). In five of six HoxA9 expressing cell lines, detectable HoxA9 expression was accompanied by higher Bcl-2 expression. Although all these lines have complex cytogenetic profiles, the two lines that also harbour 11q23 rearrangements, MV4;11 and THP-1, both had elevated HoxA9 and Bcl-2 expression. MV4;11 also carries a FLT3-ITD mutation. HL-60 cells also had elevated Bcl-2 expression, which may also be driven by c-Myc amplification. We tested the sensitivity of selected lines with elevated Bcl-2 expression to BH3-mimetic ABT-199, and used U937 lymphoma cells with reduced Bcl-2 expression as a comparison (Figure 6B). Cells were treated with 1μM ABT-199 and viability was determined after 48 hours (Figure 6B). MV4-11, THP-1, and HL-60 cells underwent apoptosis in response to the Bcl-2 inhibitor. As previously observed in HoxA9 FDMs (Figure 5D), this result suggests that Bcl-2 a required for the survival of HoxA9^High^ cells and its inhibition has significant impact on cell viability.

Recently, Valk et al studied the gene expression profile in 285 human AML patient samples [29], which were subsequently categorized into 16 subgroups based on expression array profiles (GSE1159) (Figure 6C and Supplemental Table S1). We therefore checked for the association between HoxA9 and Bcl-2 expression in these publically available expression arrays. Of the subgroups suggested by Valk et al, those with 11q23 rearrangements or FLT3-ITD mutations had elevated HoxA9 and Bcl-2 expression levels (FDR p< 0.05). Thus, even in a diverse human population of cytogenetically complex disease, the association between elevated HoxA9 and Bcl-2 expression could be observed. This was complemented by analysis of a second array dataset that compared silenced to control HoxA9 expression in AML cell lines with 11q23 rearrangements (GSE13714) (Supplemental Figure S4A). Bcl-2 expression was significantly reduced in cells expressing each of 2 independent HoxA9 short hairpin constructs, compared to control constructs (FDR p<0.05). To directly test whether MLL-rearrangements could regulate HoxA9 and Bcl-2 expression, we infected murine hematopoietic progenitor cells with lentivirus encoding MLL-AF9 under the control of a doxycycline-repressible promoter [30]. In the presence of MLL-AF9, both HoxA9 and Bcl-2 expression were elevated. When MLL-AF9 expression was repressed, HoxA9 and Bcl-2 expression declined (Figure 6D). These data emphasize the direct correlation between HoxA9 and Bcl-2 expression, both in established human AML cell lines and in patient samples. This appears to be strongest in AML with 11q23 rearrangements and/or FLT3-ITD mutations.

### Bcl-2 deletion delays AML progression

**Figure 7:**
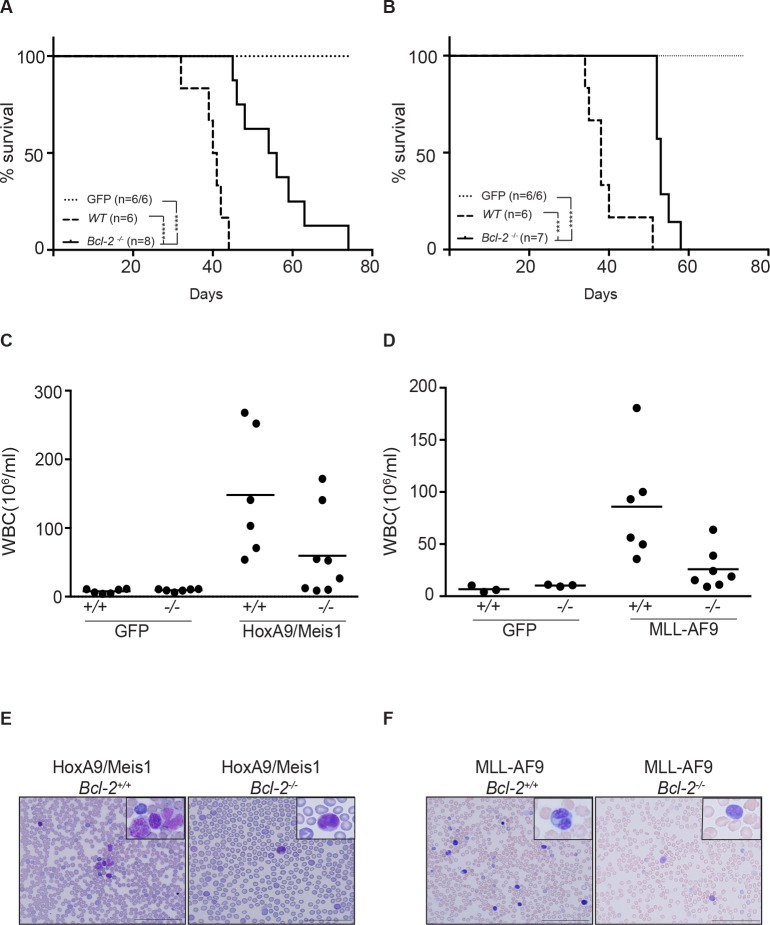
Loss of Bcl-2 expression delays AML models *in vivo* (A-B) Survival of AML-burdened mice after transplantation with HoxA9/Meis1- (left panel) or MLL-AF9- (right panel) transformed cells of wild-type (filled lines) and *Bcl-2*^−/−^ (dashed lines)genotypes. Number of mice used in each group indicated in brackets (wt/*Bcl-2*^−/−^). GFP control mice (dotted lines) from the same experiment did not develop disease independent of the genotype, thus confirming the potential of engraftment of *Bcl-2*^−/−^. (***) P < 0.0005; (****) P <0.0001. All mice transplanted with *Bcl-2*^−/−^ transformed AML cells presented a low white blood cell count compared to wild-type transplanted mice. Shown are the total numbers of white blood cells in peripheral blood ±SEM of mice bearing HoxA9/Meis1 (C) or MLL-AF9 (D) induced AML of the indicated genotype; (E and F) Representative data of the proportion of cells with morphologic features of blasts following May Grunwald Giemsa staining of blood smears preparation. Magnification 40x or inset 100x. Scale bar 100μm.

To determine whether Bcl-2 expression plays a role in HoxA9-dependent leukemias *in vivo*, hematopoietic progenitor cells from C57BL6/Ly5.2 *Bcl-2*^+/+^(wt) or *Bcl-2*^−/−^ embryos were transduced with retrovirus expressing HoxA9/Meis1, MLL-AF9 or GFP and then transplanted into irradiated C57BL6/Ly5.1 recipients. All mice that received *Bcl-2*^+/+^ cells transduced with HoxA9/Meis1 or MLL-AF9 developed rapid and aggressive leukemia with an average latency of 40 days (Figure 7A and B). Transplantation of GFP infected cells resulted in engraftment and reconstitution of normal hematopoiesis. Transplantation of *Bcl-2*^−/−^ cells significantly delayed the development of leukemias for up to 30 days (Figure 7A and B). Interestingly, there was a significant reduction in white blood cell count of *Bcl-2*^−/−^ reconstituted mice compared to wild-type, even as mice became sick (Figure 7C and D). The majority of animals transplanted with *Bcl-2*^−/−^ cells presented low blast cell counts in peripheral blood (Figure 7E and F). These results show that Bcl-2 expression contributes to the leukocytosis in these models of AML and is an important variable determining the disease severity.

**Figure 8:**
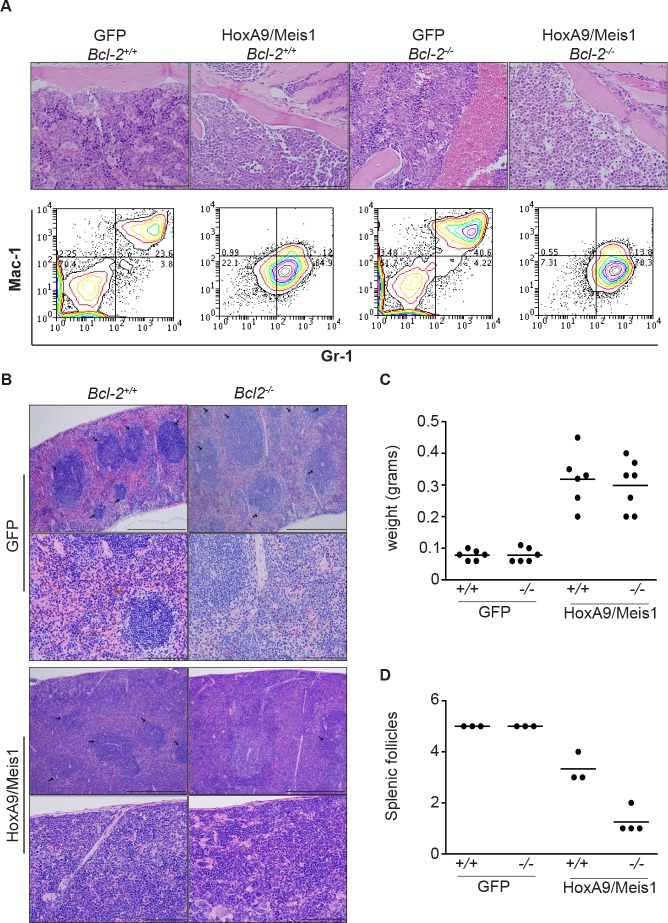
AML transplantation of Bcl-2-defcient cells induced destructive disease in spleen (A) Histological comparison of bone marrow from GFP and mice burdened with HoxA9/Meis1-induced AML of wild-type and *Bcl-2*^−/−^. Magnification 20x, scale bar 200μm. The top panels show the presence of leukemic blasts in HoxA9/Meis1 symptomatic (anemia, splenomegaly, elevated leukocytes count) mice. The bottom panels are representative flow cytometry analysis of Mac-1 and Gr-1 expression in bone marrow cells. Data show a uniform Mac-1/Gr-1 positive population in cells from HoxA9/Meis1 AML independent of the genotype. (B) Histological examination of spleens from GFP control and mice burdened with HoxA9/Meis1 AML. Hematoxylin and eosin stained sections show presence of blasts in spleens of mice transplanted with wild-type and *Bcl-2*^−/−^ HoxA9/Meis1, confirming AML development in both genotypes. Top panels of GFP and HoxA9/Meis1 magnification 10x, scale bar 500μm; lower panels, magnification 40x, scale bar 100μm. Despite the presence of blast cells and similar profile of splenomegaly (C), mice transplanted with *Bcl-2*^−/−^ HoxA9/Meis1 cells presented fewer splenic follicles (B and D). (D) Data represent splenic follicles score values from the indicated genotypes. Splenic follicles were blinded score from 1 to 5 with the maximum score being applied to spleens presenting splenic follicles as high as GFP control mice. Analysis was performed in 3 sections from each mouse.

*Bcl-2*^−/−^ transplanted mice developed a disease characterized by diminished leukocytosis, but as in wild type mice, developed splenomegaly and bone marrow infiltration. We analyzed H&E stained sections of bone marrow and spleen, and used flow cytometry and immunostaining to compare expression of surface antigens in bone marrow derived AML cells. Bone marrow from wild-type and *Bcl-2*^−/−^ HoxA9/Meis1 mice displayed similar phenotypic characteristics, with the marrow consisting almost entirely of blasts with uniform expression of Mac-1 (CD11b) and Gr-1 (Figure 8A), typical signs of AML. The spleens of wild-type and *Bcl-2*^−/−^ engrafted mice were enlarged (Figure 8B and C). However, we observed a clear morphological distinction between the splenic architecture of wild-type and *Bcl-2*^−/−^ AML. Wild-type AML spleens had similar numbers of splenic follicles as GFP mice. In contrast, *Bcl-2*^−/−^ AMLs had significantly fewer splenic follicles, indicative of a more destructive local disease (Figure 8A and D). Thus, the AML caused by transplantation of Bcl-2-deficient cells was characterized by delayed onset, significantly lower peripheral white cell counts and more destructive disease in organs such as the spleen. This suggests that Bcl-2 is required for survival of leukemic cells in peripheral blood, but not in the microenvironment of the bone marrow or spleen.

## DISCUSSION

HoxA9 overexpression is a feature of leukemia with MLL rearrangements, trisomy 8 [7] and the t(7:11)(p15:p15) translocation that fuses the homeodomain of HoxA9 to Nup98 [8]. The leukemogenic effects of HoxA9 overexpression include promoting leukemic cell self-renewal and inhibiting differentiation [9-12]. Here we show that HoxA9 overexpression maintains expression of Bcl-2, which, *in vitro*, is important for HoxA9-dependent immortalization, and *in vivo*, permits the full manifestation of AML, in particular the survival of AML cells in the peripheral circulation.

This specific role for Bcl-2 in myeloid cell immortalization and AML progression adds to the work that established Mcl-1 as the critical anti-apoptotic Bcl-2-like protein in AML [21]. Deletion of Mcl-1 prevents engraftment in several AML models and prevents development of AML in secondary recipients. However, in this study, conditional deletion of Bcl-2 was not tested. Using similar models to Glaser et al, we find that Bcl-2 expression has a role in the progression of AML, but not in the development of the disease. Accordingly, higher Bcl-2 expression is associated with increase severity, higher peripheral white cells counts and shorter survival of HoxA9-driven AML. In human AML, there is an association between elevated Bcl-2 expression and increased white cell counts [31], however it is not yet known to what extent this is driven by MLL-rearrangements or HoxA9.

Our results indicate that AML, characterized either by elevated HoxA9 expression, MLL-rearrangements or elevated Bcl-2 expression, may be treated with drugs such as ABT-737 or the more specific Bcl-2 antagonist, ABT-199, that target anti-apoptotic Bcl-2 family members. Human AML cells lines with 11q23 rearrangements (in addition to many other genetic alterations) were, to greater or lesser degrees, killed by ABT-737, as has been previously characterized [32]. However, our *in vivo* models provide an important consideration in the use of these agents in AML. Bcl-2 deletion delayed disease progression, probably because the peripheral blast count was reduced. However, this did not prevent the progression of AML within organ niches such as the spleen and bone marrow. Thus, whilst it is the case that Bcl-2 family proteins have a significant influence on the response of AML to chemotherapy, at least in an *ex-vivo* environment [32], one might anticipate that targeting Bcl-2 specifically would be useful primarily to reduce peripheral white blood cell counts in patients with significant leukocytosis and consequently delay the progression and severity of the disease.

Despite the complexity of genetic lesions that will invariably be present in any population of patients with AML [33], we observed a correlation between elevated HoxA9 and Bcl-2 expression, particularly in association with 11q23 rearrangements or the expression of FLT3-ITD. This subgroup of AML (and ALL) may be the patients most likely to gain some benefit from drugs like ABT-263 and ABT-199, which may reduce leukocytosis and delay disease progression. However, it is clear from our results and Glaser et al [21] that Mcl-1 is the principal Bcl-2 family member to target for inhibition to achieve complete clearance of AML.

The diminished numbers of peripheral *Bcl-2*^−/−^ blasts compared to those resident in the spleen and bone marrow may result from cell autonomous differences between these cell populations. Mcl-1 is absolutely required for the survival of the population of leukemic cells that permit serial transplantation of AML [21], the so-called leukemic stem cells. The peripheral AML blast population may contain significantly fewer such cells, but instead is enriched for cells with a specific requirement for Bcl-2 to maintain survival. Alternatively, host dependent factors may be more important. The bone marrow and splenic niche may provide abundant survival signals, including cytokines such as GM-CSF and IL-3 that maintain expression of Mcl-1 [34,35]. The absence of these signals in peripheral blood would result in a decline in Mcl-1 levels and perhaps drive selection in favor of cells that maintain expression of other Bcl-2 proteins, whose levels are less dependent on external survival signals.

The association between HoxA9 and *Bcl-2* expression has been previously observed. *HoxA9*^−/−^ mice have reduced numbers of bone marrow myeloid and lymphoid progenitor cells [2] that may result from increased apoptosis of lineage-committed progenitor cells as well as defects in maturation. This is supported by the findings of increased apoptosis and decreased Bcl-2 expression in the fetal thymus of *HoxA9*^−/−^ mice [3]. Definitive evidence of direct regulation of Bcl-2 by HoxA9 would require to localization of transcriptional complexes that include HoxA9 in conserved regulatory regions of *Bcl-2*. However many other transcription factors, including c-Myc and Myb are also known to promote Bcl-2 expression [36,37], and are also upregulated in HoxA9-immortalised cells [17]. Thus HoxA9 may promote *Bcl-2* expression indirectly via these transcriptional regulators. Our results and others [11] show that HoxA9-Pbc interactions are required for HoxA9 function. Nup98-HoxA9, on the other hand, no longer requires Bcl-2 for efficient immortalization of hematopoietic progenitor cells. Nup98-HoxA9 also does not require an intact Pbc-interaction motif to promote immortalization or leukemia [38]. It will be interesting and informative to compare the genes directly regulated by HoxA9 and Nup98-HoxA9. Our results would predict that *Bcl-2* would be among the differently regulated genes.

In conclusion, we have identified Bcl-2 as a key protein mediating HoxA9-dependent immortalization. This adds Bcl-2-regulated apoptosis to the pathways that contribute to the oncogenic effects of HoxA9 overexpression. Deciphering the molecular links between HoxA9 and the regulation of Bcl-2 family members will greatly expand the potential therapeutic strategies that may be used in malignancies in which HoxA9 overexpression plays a key role.

## MATERIAL AND METHODS

### Mice and cell lines

Mice (*Bcl-2*^−/−^, *Bax*^−/−^;*Bak*^−/−^, *Mcl-1*^fl/fl^, *Bcl-x*^fl/fl^, *Bcl-2*^fl/fl^ and Rosa26-Cre ERT2) from which IL-3- or GM-CSF-dependent cell lines were derived, have been previously described [19-23,39,40]. Cells constitutively expressing IL-3 were generated by co-culture of HoxA9 FDM cells with fibroblasts (ψ2 cells) expressing IL-3 from a retrovirus (kind gift from Suzanne Cory). Cells were tested for endogenous IL-3 expression by determining their survival in liquid culture and soft agar in the absence of exogenous IL-3. All FDM lines were routinely cultured at 37°C in a 10% CO_2_ humidified atmosphere in DMEM low glucose media (Invitrogen) supplemented with 10% fetal calf serum, 50nM 4-OHT or 1ug/ml Doxycycline (Sigma) and 0.5ng/ml IL-3 and/or 2.5ng/ml GM-CSF (Peprotech). 293T cells and fibroblasts (Ψ2) cells were cultured in DMEM/10% FCS.

### Cloning and lentiviral production

*M.musculus* Flag-HoxA9 was cloned into the MSCV-GFP retroviral vector and lentiviral inducible vectors pF 5xUAS SV40 puro GEV16 or pFTREtight MCS rtTAadvanced GFP (doxycycline inducible). The hexapeptide-mutant Flag-HoxA9 was cloned into the lentiviral inducible vector pF 5xUAS SV40 puro GEV16. MSCV-Nup98-HoxA9 retroviral vector was previously described [38] as was HoxB8 lentiviral inducible vector [41]. Retrovirus and lentivirus production are detailed in supplemental material and methods.

### Generation of FDM lines

IL-3- or GM-CSF-dependent myeloid cell lines overexpressing Flag-HoxA9, HoxB8 or Nup98-HoxA9 were generated by retroviral or lentiviral transduction of hematopoietic progenitors from E14.5 fetal livers or bone marrow stem cells with the different constructs using a Retronectin-based protocol. MLL-AF9 tetracycline-repressible cells were generated by retroviral transduction of myeloid progenitor cells from E14.5 fetal livers with pMSCV-IRES-AtTA and pSIN-TREtight-Red2-ires-MLL-AF9 (a kind gift from Luke Dow and Scott Lowe). Three weeks after infection dsRed cells were sorted in culture in IMDM media containing 10%FCS and 5ng/ml IL-3. Infection and selection of cell lines are detailed in supplemental material and methods.

### Generation and culture of leukemic cells

All in vivo experiments were conducted in accordance with the guidelines of The Walter and Eliza Hall Institute Animal Ethics Committee. HoxA9/Meis1 retroviral construct and MLL-AF9 retroviral construct were previously described [42,43]. Viral supernatants were produced in 293T cells by co-transfection of expression constructs and packaging plasmids. Fetal liver cells (E14.5) from C57BL/6 Ly5.2 mice were infected with viral supernatant using the retronectin protocol. Transduced cells were cultured in alpha-MEM medium (Invitrogen) supplemented with 10% FCS, 2mM L-glutamine, 100ng/mL murine stem-cell-factor (Peprotech), 10ng/mL IL-6, 50ng/ml TPO and 10ng/ml Flt3 (WEHI). After two rounds of infection, cells were injected into sub-lethally g-irradiated (7.5 Gy) C57BL/6 Ly5.1 mice. Mice were collected when disease was evident. Parameters used to determine leukemia were weight-loss, enlarge spleen, anemia, lethargy and hunched posture. Leukemic cells were obtained from bone marrow of sick mice. Cells were cultured at 37°C in a 10% CO_2_ humidified atmosphere in IMDM media supplemented with 10% fetal calf serum and 5ng/ml IL-3.

### Cell cycle, proliferation and apoptosis analysis

Cell viability was determined by flow cytometry analysis of Annexin V-FITC/PI stained cells, as previously described [44]. Cell cycle analysis of total DNA content was performed as described [45] and analyzed using the ModFit software. Cell proliferation was determined by flow cytometric cell counts or trypan exclusion. Detailed methodology is described in supplemental material.

### Western Blot and reagents

Cells were lysed in sodium dodecyl sulfate (SDS) buffer (50mM Tris-HCL, pH 6.8, 2% SDS, 10% glycerol and 2,5% β-mercaptoethanol) and boiled for 10 min. The antibodies used were; Anti-mouse Bcl-2 mAb (BD Biosciences), anti-Actin mAb and pAb anti-Bax and anti-Bak (Sigma), anti-Bad pAb (Cell Signaling), anti-Bim pAb (Stressgen), anti-Puma pAb (ProSci), anti-HoxA9 pAb and anti-MLL clone N4.4 mAb (Millipore), anti-Mcl-1 pAb (Rockland) and anti-Bcl-x_L_ pAb (R&D Systems). pAb anti-Bid, anti-Bmf and anti-A1 pAb were a kind gift from Andreas Strasser, Lorraine O'Reilly and Marco Herold respectively. ABT-737 and ABT-199 was a kind gift from David Huang supplied by AbbVie [46,47].

### Clonogenic assays

Clonogenic assays were performed as previously described [48].

Detailed methodology is described in supplemental Material and Methods.

## Supplemental Methods, Figures and Table


