# Supplementary material for: HoxA9 regulated Bcl-2 expression mediates survival of myeloid progenitors and the severity of HoxA9-dependent leukemia
Source: Oncotarget. 2013 Sep 15;4(11):1933–47. doi: 10.18632/oncotarget.1306 (PMC3875760; doi:10.18632/oncotarget.1306)
Supplement: Supplementary file 1 [file oncotarget-04-1933-s001.doc]

**SUPPLEMENTAL MATERIAL AND METHOD**

**Cloning and Lentiviral production**

The pFTREtight MCS rtTAadvanced GFP vector was generated by subcloning the tetracycline response elements from pTRE3G_pGK3G_GFP into pF5xUAS GEV16-EGFP using PacI and AscI. HoxA9, HoxB8 and Nup98-HoxA9 were subcloned into this new vector. HoxB8 4-OHT inducible lentiviral vector and doxycycline inducible vector were previously described [41].

Murine HoxA9 was amplified by PCR from pMSCV IRES-GFP Flag- HoxA9 (Addgene #8515) using oligonucleotides #1 (5’ GCACCGGTCCGCCATGGACTACAAGGACGACGATGACAAG 3’) and #2 (5’ GCGCTAGCAAGCTTACAATACCTCCTCCATCA 3’). Oligonucleotide #1 introduced unique restriction site *AgeI*, 5’ to the start codon of HoxA9 and oligonucleotide #2 introduced unique restriction site *NheI* 3’ to the stop codon. HoxA9 fragment was cloned into *AgeI- NheI* digested pF 5xUAS SV40 puro GEV16 vector. *BamHI*- *NheI* HoxA9 fragment obtained from PCR amplification using forward primer (5’GCGGATCCCCGCCATGGACTACAAGGACGACGATGACAAGATGGCCACCACCGGGGCC 3’) and oligonucleotide # 2 was cloned into the doxycycline inducible lentiviral vector, pFTREtight MCS rtTAadvanced GFP.

Hexapeptide mutant Flag-HoxA9 fragment was obtained using previously described oligonucleotides (#1 and #2) and oligonucleotide 5’CGGCTGCCAACgcagctgcaGCTCGCTCCAC 3’. Tryptophan, Leucine and Histidine were replaced by alanine as shown by the underlined sequence using PCR mutagenesis.

Nup98-HoxA9 was amplified from MSCV-retroviral vector using forward primer 5’ GCGGATCCGAATTCCCGCCATGGACTACAAGGACGACGATGACAAGATGTTTAACAAATCA 3’ and reverse primer 5’ GCGCTAGCGAATTCTCTAGATCACTCGTCTTTTGC 3’ and cloned into the doxycycline inducible vector pFTREtight MCS rtTAadvanced GFP.

Lentivirus was produced by transient transfection of 2x10^6^ HEK-293T cells in a 10cm dish with 2.5μg of packaging vector, pCMV δR8.2, 1μg of envelope vector, pMD2G- VSVG and 1.5μg of pF 5xUAS SV40 puro GEV16 or pFTREtight MCS rtTAadvanced GFP, using Effectene (Qiagen). Media was changed the following day and cells left for 24 hours before the supernatant was filtered. Lentivirus was used immediately or stored at -80°C. Viral titers were performed by infecting known numbers of FDC-P1 cells at varying dilutions of virus-containing supernatant, selecting infected cells with puromycin (0.5 μg/ml) and determining the viability. Assuming that 1 active viral particle maintains the viability of 1 cell in puromycin, the MOI was determined by the number of viable cells per ml of viral supernatant used.

Retrovirus was produced as described for lentivirus using 1.5μg of packaging vector (gag), 1μg of envelope vector (env) and 2.5μg of retroviral vector pMSCV Neo (Nup98-HoxA9) or GFP (Flag- HoxA9)

**Generation of FDM lines**

Factor-dependent myeloid cell lines overexpressing Flag-HoxA9, HoxB8 or Nup98-HoxA9 were generated by isolating c-kit positive, lineage negative, hematopoietic progenitors from E14.5 fetal livers using an antibody cocktail mix of c-kit-APC, Gr-1-FITC, Sca-1-PE, NK1.1-FITC, B220-FITC and TER-119-FITC (BD Pharmingen). Cells were incubated for 45 min at 4°C in PBS/2%FCS containing the antibody mix, washed twice in PBS/2%FCS and resuspended in PBS/2%FCS/Propidium Iodide (PI) (10μg/ml). C-kit positive/PI negative cells were isolated by Flow Cytometry and cultured for 24h at 37°C in a 10% CO_2_ humidified atmosphere in low glucose DMEM media supplemented with 10% fetal calf serum (FCS: JRH Laboratories), 25ng/ml Stem Cell Factor (Invitrogen) and 5ng/ml of Interleukin 3 (Peprotech). Lentiviral supernatant was used to infect cells via spinoculation at 30°C for 90 minutes at 2500rpm in the presence of 5µg/ml polybrene (Sigma). Twenty-four hours after infection, cells were replated in low glucose DMEM supplemented with 10% FCS, 0.5ng/ml or 5ng/ml IL-3, 25ng/ml Stem Cell Factor and 50nM 4-OHT (Sigma) for induction of HoxA9 expression. Three days after 4-OHT addition, cells successfully expressing HoxA9 were selected with 0.5µg/ml of puromycin (Sigma) for 10 days in low glucose DMEM media containing 10% FCS, 0.5ng/ml or 5ng/ml IL-3, 2.5ng/ml or 10ng/ml GM-CSF (Peprotech) and 50nM 4-OHT.

For generation of FDM cells using retroviral transduction, hematopoietic progenitors from E14.5 fetal livers or bone marrow stem cells were infected with the different constructs using a retronectin-based protocol. Twelve-well plates (non-treated polystyrene- Corning) were coated for 16h at 4**°**C with 32μg/ml of retronectin. The next day, 1ml of viral supernatant was added to wells and spun at 3500rpm for 1h. Cells were added to wells containing 200μl of virus and incubated for at 37**°**C, 10% CO2. After 2 rounds of infection cells were cultured as previously described in DMEM or IMDM media containing 10%FCS, 5ng/ml IL-3 and 10ng/ml GM-CSF. Cells were sorted for GFP expression or selected with G418.

**Cell proliferation**

In all proliferation assays, cells were passaged every third day and dilution of cells taken into consideration when determining total cell number. At every cell count, cells were replated in liquid culture at same cell numbers as day 0. Final cell numbers were determined by cell number counted x dilutions at each cell passage. Flow cytometric cell counts were performed by resuspending cells in Annexin-V-FITC/PI buffer containing 1:10 dilution of known concentration of fluorescent microsphere beads (Beckman Coulter). A minimum of 10000 cells was acquired and the total cell number was determined by the number of viable cells acquired divided by number beads/µl detected. Trypan-blue exclusion cell counts were performed by resuspending cells 1:10 in 0.2% trypan blue solution and negative cells counted (Invitrogen Countess). Total cell number was determined considering initial trypan blue dilution and dilution at each cell passage.

**Clonogenic assays**

Growth factor and/or HoxA9 deprivation assays were performed by removal of cytokines and/or 4-OHT from FDM cells by washing cells several times in PBS. At the indicated time points, known numbers of cells were plated in low glucose DMEM/ 20% FCS/ 0.3% agar in the presence or absence of IL-3 or GM-CSF and 0.5µM 4-OHT as indicated. After 15 days the number of colonies was determined and expressed as colonies per 1000 cells plated. Relative clonogenicity was calculated as previously described [48].

**Surface antigen staining**

Expression of surface antigen was determined by staining 1-2x10^5^ cells with the following fluorochrome conjugated antibodies, c-kit-APC, Sca-1-PE, CD11b-PE, Gr-1-FITC, NK1.1-FITC, B220-APC, TER-119-FITC, CD135-PE, CD34-FITC (all from BD Biosciences), F4/80-PE (AbD Serotec), CD123-PE and CD131-PE (R&D). Live cells were analyzed by flow cytometry using FACSDiva (Becton Dickinson).

**Reverse Transcription and Real-Time Polymerase Chain Reaction**

RNA was extracted from a minimum of 5x10^6^ cells using RNeasy RNA extraction kit (Qiagen) and 1.5µg of RNA was reverse transcribed using H-HLMV and random primers (Promega). Levels of Bcl-2 mRNA were quantified using the Universal Probe Library (Roche) and Faststart Taqman Probe Mastermix (Roche Diagnostics.

The probe mixes and primers used for Bcl-2

Bcl-2, probe mix #75, forward primer 5’ gtacctgaaccggcatctg 3’ and reverse primer 5’ ggggccatatagttccacaa 3’

Experiments were run on an ABI 7900 HT instrument (Applied Biosystems, Foster City, CA), with the following cycling parameters; 95ºC for ten minutes to activate the polymerase followed by 40 cycles at 95ºC for 15 seconds and 60ºC for one minute in a two-step thermal cycle. All samples were normalised to the housekeeping genes Sdha (probe #108), 5’ cagttccaccccacaggta 3’ and 5’ tctccacgacacccttctg 3’ and Polr2a (probe #95), 5’ aatccgcatcatgaacagtg 3’ and 5’ tcatccattttatccaccacct 3’. Results were analysed using the LightCycle 480 software.

**Bioinformatics analysis on public available microarray datasets and ChIP-sequencing**

Microarray re-analysis

569 ALL (including 4 normal CD34+ and 4 CD10+CD19+) and 285 AML (including 5 NBM and 3 CD34+) were downloaded from the Array Express database (E-GEOD-28497) and GEO database (GSE1159) [29] databases respectively. Microarray datasets for the 2 most effective HOXA9 shRNA and the GFP-control shRNA in MOLM14 (MLL AML) were downloaded from GEO GSE13714 [18]. The raw microarray data were normalized using RMA. Quality control of the array data was assessed using the Normalised Unscaled Standard Errors (NUSE) plot. The differential gene expression was performed using the linear modeling and the empirical eBayes moderated t statistics (LIMMA) [49] incorporated with the array quality weights [50]. The false discovery rate (FDR) was controlled using the Benjamini-Hochberg method. For all the comparisons that were performed for ALL and AML gene expression in the leukaemia samples was compared to the normal samples. Only the genes with FDR<0.05 were considered as statistically significant.

Hoxa9/Meis1 ChIP-sequencing data analysis on Bcl2 locus

The Wig files for murine and human HoxA9 and Meis1 were downloaded from NCBI GEO accession number GSE38339 and GSE33518 respectively. Peak enrichment regions were visualized using the UCSC genome browser. MEME-ChIP was used to discover common patterns in mouse and human HOXA9 binding peak sequences at the Bcl2 locus.  TGACAG was identified as one of the most common patterns in these sequences (E value = 0.039). We then subjected this motif to the motif comparison tool TOMTOM for comparison against a JASPAR and UniPROBE database of known motifs identifying Meis1 (p-value=9.2x10^-6^) as a significant motif match. To enable visualization of the Human HOXA9 and MEIS1 (GSE38339) ChIP peaks aligned to the mm8 tracks for Bcl2, the human genome coordinates (hg18) were converted to mouse (mm8) using the liftOver function implemented in the UCSC genome browser.  The mouse, and the converted human HOXA9 and MEIS1 Wig files, were uploaded to the UCSC genome browser as mm8 tracks, and peaks at the Bcl2 locus are shown.  Cross-species conservation between mouse, rat and human is shown by the conservation track (green peaks).  The Hoxa9 binding sites at the Bcl2 locus are boxed. No binding sites were observed in the promoter region of Bcl2 (not shown).

**SUPPLEMENTAL FIGURE LEGENDS**

**Supplementary Figure S1**. **HoxA9 overexpression is required for proliferation and survival of myeloid progenitor cells. (A)** Myeloid progenitor cells from fetal livers were transduced with GFP, Flag-HoxA9 or Flag- hexapeptide mutant HoxA9 and cultured in soft agar in the presence of the IL-3 (5ng/ml) and GM-CSF (10ng/ml). After 14 days, the number of colonies was determined and the results expressed as colonies per 1000 cells plated. Results are means ±SEM of 3 independent experiments using 3 independent pools. **(B)** Kinetics of IL-3 and GM-CSF withdrawal in HoxA9 FDM cells. Cells were cultured in the absence of the indicated cytokines for the indicated periods. Cell viability was determined by flow cytometry by AnnexinV-FITC staining and PI exclusion. Results show the means ± SEM of 3 independent experiments using 3 independent pools and clones from each condition (IL-3 or GM-CSF). **(C)** HoxA9 FDM cells immortalized in the presence of high concentrations of cytokines IL-3 (5ng/ml) and GM-CSF (10ng/ml) were cultured in soft agar in the presence or absence of 4-OHT (50nM). Colonies numbers were counted after 14 days and results expressed as colonies per 1000 cells plated. Results are means ±SEM of 2 independent experiments using 3 independent clones of HoxA9 infected cells. **(D)** Comparison of surface markers in HoxA9-infected FDM cells. Cells were stained with antibodies against the indicated surface antigens and the percentage of expressing cells (compared to unstained controls) determined by flow cytometry at the indicated days after 4-OHT withdrawal. Results shown are means ±SEM from 4 independent pools in 3 independent experiments.

**Supplementary Figure S2. Downregulation of HoxA9 does not sensitize myeloid progenitors to chemotherapeutic drugs.** **(A)** HoxA9 FDM cells were cultured in GM-CSF and the absence or presence of 4-OHT for 48h followed by treatment with Cisplatin (20μg/ml), Etoposide (10μg/ml), SAHA (2.5μM) or cytokine withdrawal for 24h. Viability was measured by flow cytometry analysis of AnnexinV-FITC staining and PI exclusion. Results are mean ± SEM of 3 clones and 3 independent pools. **(B)** The same cells described in (A) were treated with the indicated doses of ABT-737 for 24 hours. Viability was determined by flow cytometry analysis of AnnexinV-FITC staining and PI exclusion. Data are mean ± SEM of 3 independent pools repeated in 2 independent experiments.

**Supplementary Figure S3. Bcl-2 protein expression is regulated and important for HoxA9 immortalization but not for HoxB8 or Nup98-HoxA9. (A and B)** Comparison of surface markers in *Bcl-2^+/+^* and *Bcl-2^-/-^* HoxB8 (A) and HoxA9 (B) infected cells. Cells were cultured in IL-3 (HoxB8) and in both IL-3 and GM-CSF (HoxA9) in the presence of 4-OHT (50nM). Cells were stained with antibodies against the indicated surface antigens and the percentage of expressing cells (compared to unstained controls) determined by flow cytometry. Results shown are means ±SEM from 3 independent *Bcl-2^+/+^* and 4 independent *Bcl-2^-/-^* pools in 2 independent experiments. **(C)** Twenty-one days after infection, *Bcl-2^+/+^* and *Bcl-2^-/-^* HoxB8, HoxA9 and Nup98-HoxA9-dependent cells were plated in 0.3% soft agar in the presence of IL-3 (HoxB8) or IL-3 and GM-CSF (HoxA9 and Nup98-HoxA9) and 4-OHT (HoxB8 and HoxA9 cells). Colonies were counted after 14 days. Results are means ±SEM of 3 independent experiments of 6 independent pools from each genotype. **(D)** Viability of 4-OHT untreated hematopoietic progenitor cells from mice with homozygous LoxP-flanked Bcl-2, Mcl-1 or Bcl-xL alleles crossed with Rosa:cre-ER infected with HoxA9 (filled bars) or Nup98-HoxA9 (unfilled bars) and cultured in IL-3 and GM-CSF. Cell death was measured by flow cytometry using Annexin V-FITC staining and PI exclusion at same time as cells in Figure 4d. Results show means ±SEM of 5 independent experiments from HoxA9 FDM cells and 4 independent experiments from Nup98-HoxA9 FDMs.

**Supplementary Figure S4. The molecular mechanisms involved in the regulation of Bcl-2 expression by HoxA9. (A)** Expression of HoxA9 (left panel) and Bcl-2 (right panel) mRNA following 44 hours transduction of MOLM-14 cells with 2 independent short hairpin RNAi constructs targeting HoxA9 or a GFP-control shRNA vector in triplicates. Expression array data is from Faber et al [18] (GSE13714). The false discovery rate (FDR) was controlled using the Benjamini-Hochberg (BH) method. **(B)** The ChIP enrichment profile for HoxA9 and Meis1 is shown for the mouse Bcl2 locus. ChIP peaks are based on the studies described by Huang et al ^17^ (GSE33518) and in GSE38339.   The HoxA9 and Meis1 Wig files were uploaded to the UCSC genome browser as mm8 tracks, and peaks at the Bcl2 locus are shown.  For the murine study there were no peaks detected across the Bcl2 locus in the control samples. The HoxA9 and Meis1 Wig files were uploaded to the UCSC genome browser as mm8 tracks, and peaks at the Bcl2 locus are shown.  Cross-species conservation between mouse, rat and human is shown by the conservation track (green peaks).  The HoxA9 binding sites at the Bcl2 locus are boxed. No binding sites were observed in the promoter region of Bcl2 (not shown). (**C**) The visualization of murine and human binding of HoxA9 and Meis1 at the Bcl2 locus based on ChIP-seq datasets. For each binding fragment bound by HoxA9, the DNA sequence was extracted and searched for the HOX (red) or MEIS1 (orange) transcription factor motif.  For each peak matching motifs are shown on the negative strand; HOX motifs match the consensus described in Pearson et al [1] and Huang et al [17]. MEIS motifs are matches to the consensus in [17]. (**D**) TGACAG was identified as one of the most common patterns in these sequences (E value = 0.039). This motif was subjected to the motif comparison tool TOMTOM for comparison against a JASPAR and UniPROBE database of known motifs identifying Meis1 (p-value=9.2x10^-6^) as a significant motif match.

**TABLE LEGEND**

**Table 1. Expression of HoxA9 and Bcl-2 in AML and ALL.** HoxA9 and Bcl-2 expression analysis from 285 AML and 569 ALL patient samples. AML samples were grouped into each of 16 clusters characterized by gene expression. Expression for each cluster is shown relative to the normal control group (NBM and CD34+, n=8). Expression data from ALL samples was based on the presence (n=28) or absence (n=541) of MLL abnormalities is shown compared to normal control samples (CD34+ and CD10+CD19+, n=8). Significant gene expression change was defined as BH-adjusted p<0.05.

**SUPPLEMENTAL FIGURES**

**Supplementary Figure S1**

**Supplementary Figure S2**

**Supplementary Figure S3**:

**Supplementary Figure S4**


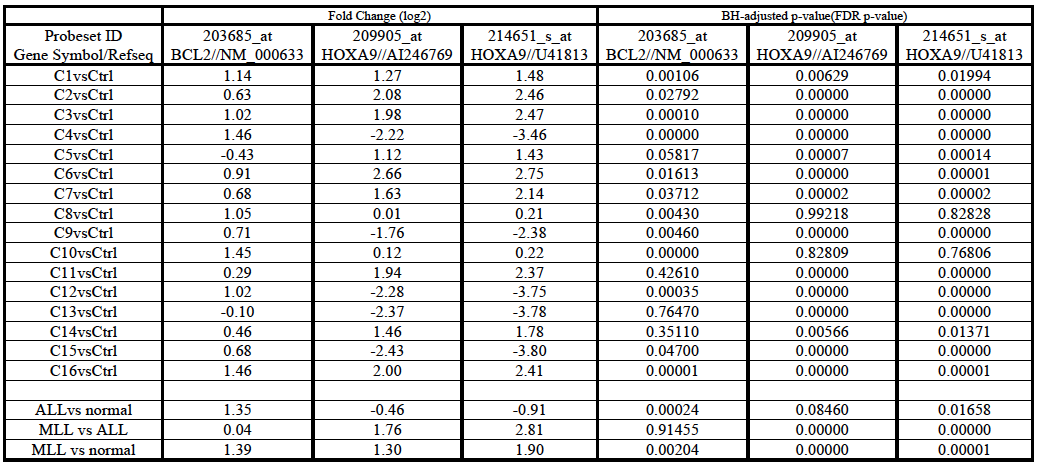
**Table 1**:
